# Correction: Sevoflurane Induces Learning and Memory Impairment in Young Mice Through a Reduction in Neuronal Glucose Transporter 3

**DOI:** 10.1007/s10571-024-01520-2

**Published:** 2025-01-16

**Authors:** Jinpiao Zhu, Zongze Zhang, Junke Jia, Lirong Wang, Qiuyue Yang, Yanlin Wang, Chang Chen

**Affiliations:** Department of Anaesthesiology, Zhongnan Hospital, Wuhan University, East Lake Road, Wuhan, 430071 Hubei China

**Correction to: Cellular and Molecular Neurobiology (2020) 40:879–895** 10.1007/s10571-019-00779-0

The original version of this article unfortunately contained an error in Fig. 4.

The histogram present (on right panel) in Fig. 4E is incorrect. This bar chart presents the statistical analysis of Glut3 immunofluorescence expression in Figure E. Quantification of GLUT3 intensity in the CA1 region of the hippocampus and the temporal lobe, measured 24 h after the final sevoflurane exposure. In both the CA1 region of the hippocampus and the temporal lobe, *P* < 0.05 for sevoflurane compared to the control group.

The correction of this image does not alter the statistical differences between groups and has no impact on the conclusions of the paper.

**Incorrect version of Fig.** 4

**Fig. 4:**
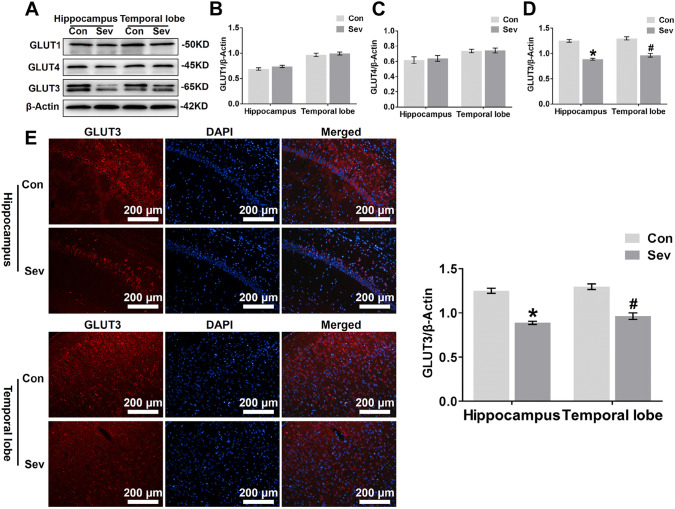
Sevofurane decreases GLUT3 protein expression in the hippocampus and temporal lobe. **a** WB analysis of GLUT1, GLUT4, and GLUT3 protein expression in the hippocampus and temporal lobe of young mice 24 h after the last exposure to sevofurane. n = 4 for each group. **b, c, d** Histograms showing the quantification of GLUT1, GLUT4, and GLUT3 blots in the hippocampus and temporal lobe. In the hippocampus, *P < 0.05 versus the Con group. In the temporal lobe, ^#^*P* < 0.05 versus the Con group. **e** Fluorescent images showing GLUT3 expression in neurons of the hippocampal CA1 area and temporal lobe (left panel). Quantifcation of GLUT3 intensity in the CA1 region of the hippocampus and temporal lobe 24 h after the last exposure to sevofurane (right panel). In the CA1 region of the hippocampus, *P < 0.05 versus the Con group. In the temporal lobe, ^#^*P* < 0.05 versus the Con group. n = 4 for each group. The data are presented as the mean ± s.e.m

**Corrected version of Fig.** 4

**Fig. 4:**
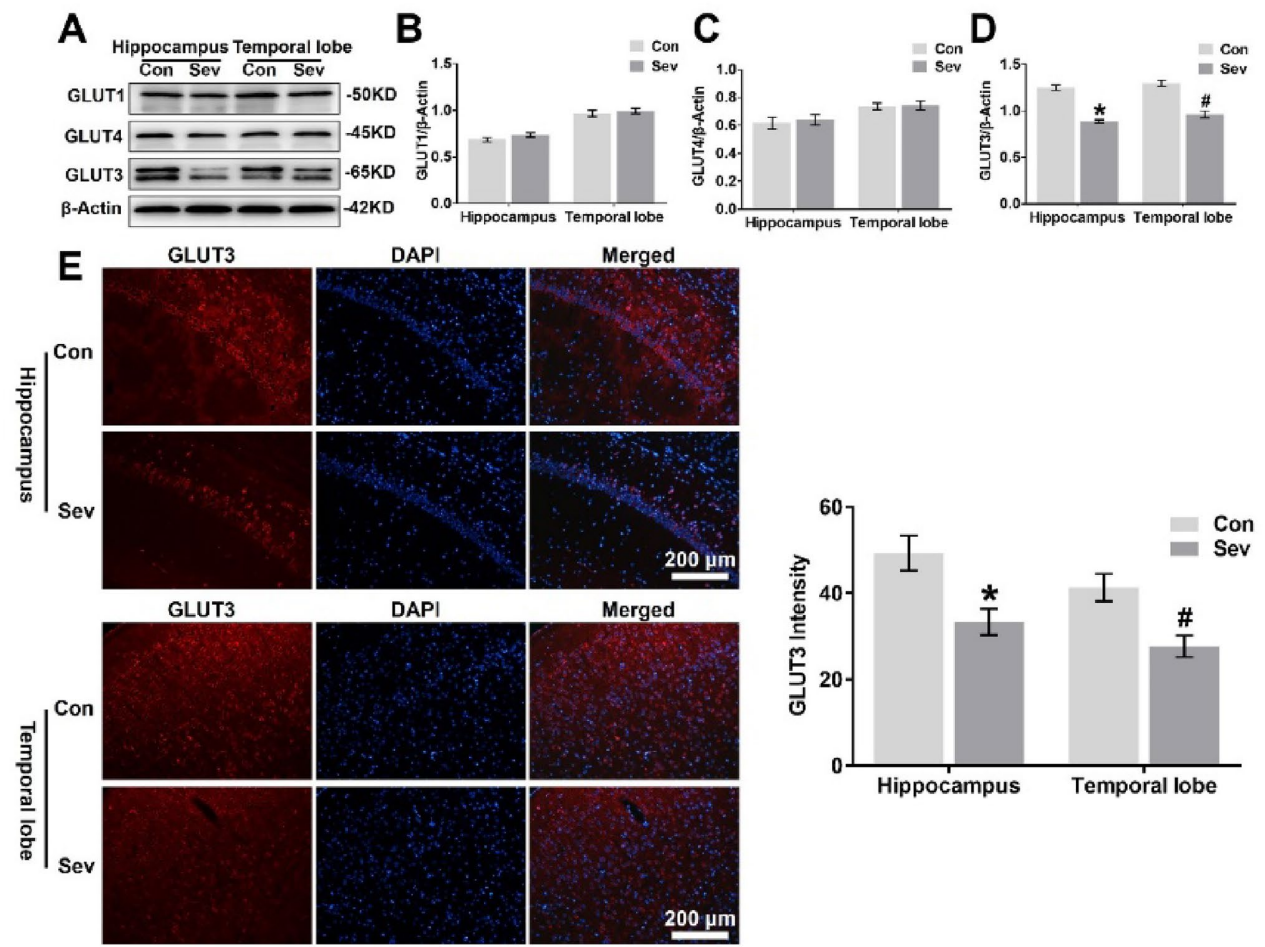
Sevofurane decreases GLUT3 protein expression in the hippocampus and temporal lobe. **a** WB analysis of GLUT1, GLUT4, and GLUT3 protein expression in the hippocampus and temporal lobe of young mice 24 h after the last exposure to sevofurane. n = 4 for each group. **b, c, d** Histograms showing the quantification of GLUT1, GLUT4, and GLUT3 blots in the hippocampus and temporal lobe. In the hippocampus, *P < 0.05 versus the Con group. In the temporal lobe, ^#^*P* < 0.05 versus the Con group. **e** Fluorescent images showing GLUT3 expression in neurons of the hippocampal CA1 area and temporal lobe (left panel). Quantifcation of GLUT3 intensity in the CA1 region of the hippocampus and temporal lobe 24 h after the last exposure to sevofurane (right panel). In the CA1 region of the hippocampus, *P < 0.05 versus the Con group. In the temporal lobe, ^#^*P* < 0.05 versus the Con group. n = 4 for each group. The data are presented as the mean ± s.e.m

The original article has been corrected.

